# A comprehensive analysis of FOX family in HCC and experimental evidence to support the oncogenic role of FOXH1

**DOI:** 10.18632/aging.203934

**Published:** 2022-03-07

**Authors:** Xiwu Ouyang, Lemeng Feng, Lei Yao, Jingyu Zhang, Yao Xiao, Guodong Liu, Gewen Zhang, Zhiming Wang

**Affiliations:** 1 Department of Liver Surgery, Xiangya Hospital, Central South University, Changsha 410008, Hunan, China; 2 Department of Pancreatic Surgery, Xiangya Hospital, Central South University, Changsha 410008, Hunan, China; 3 Department of Biliary Surgery, Xiangya Hospital, Central South University, Changsha 410008, Hunan, China

**Keywords:** FOX, HCC, FOXH1, prognosis, cell invasion

## Abstract

Hepatocellular carcinoma (HCC) remains the second leading cause of cancer related deaths worldwide. Understanding about the molecular biology of HCC and development of targeted therapies are still the main focuses of this type of disease. Here, by connecting the expression levels of FOX proteins with their associated clinical characteristics using TCGA LIHC dataset, we found that 27/40 FOX proteins were highly expressed in HCC tumors compared to normal liver tissues and their expression levels were tightly associated with HCC tumor stage, tumor grade and overall survival. Our experimental results also confirmed that FOXH1 indeed played an oncogenic role in HCC development by promoting cell growth and cell migration/invasion. Mechanistic dissection demonstrated that FOXH1-induced cell growth and cell migration/invasion relied on mTOR signaling because inhibition of mTOR signaling by rapamycin could attenuate FOXH1-mediated phenotypic alterations of HCC cells. The results from orthotopic mouse model also validated that FOXH1 promoted HA22T tumor growth via triggering mTOR activation. Overall, this study not only comprehensively examines the clinical values of FOX proteins in HCC but also provides experimental evidence to support the role of FOXH1 in HCC development, building rationale to develop more effective therapies to treat HCC patients.

## INTRODUCTION

Hepatocellular carcinoma (HCC) is the most common primary hepatic cancer with an estimated 0.8 million incident cases in 2018 [1]. The majority of HCC occurs in people with either hepatitis B/C infection or aflatoxin exposure [2, 3]. Although current therapies including surgical dissection, liver transplantation, radiation therapy, targeted therapy and immune therapy improve patients’ lives a lot [4], the mortality of HCC is still very high, with an estimated 0.7 million deaths in 2018, and is responsible for the second leading cause of cancer associated deaths worldwide [1, 5]. Therefore, there is an urgent need to develop novel therapeutic strategies for better treatment of HCC patients.

As transcription factors, Forkhead Box (FOX) proteins are involved in various biological events such as proliferation, apoptosis, DNA repair and metabolism either alone or together with other transcription factors/cofactors [6]. Numerous studies have proved that the dysregulation of FOX proteins was tightly associated with cancer initiation, progression, and drug resistance [6–9]. For instance, the expression level of FOXC1 was significantly increased in HCC and it functioned as an independent predictor for HCC survival and tumor recurrence. FOXC1 promoted HCC tumor metastasis via transactivating Snail [10], a central transcription factor regulating a bundle of metastasis-related genes. Besides, the oncogenic roles of FOXG1, FOXO3, FOXK1 and FOXM1 in HCC carcinogenesis have also been recognized in recent years [11–15], suggesting that FOX proteins may be the potentially therapeutic targets of HCC. However, few efforts have been taken to comprehensively evaluate the clinical values of FOX proteins and to unfold the role of FOXH1 in HCC.

The role of mTOR (mammalian target of rapamycin) signaling has been well documented in cancer development including HCC [16, 17]. The common subunits of mTOR complex at least include the mTOR kinase, mLST8 (the mammalian lethal with SEC13 protein 8), DEPTOR (DEP Domain Containing MTOR Interacting Protein), Tel2 (telomere maintenance 2) and Tti1 (Tel2-interacting protein 1) [18, 19]. RAPTOR in mTORC1 and RICTOR in mTORC2 make these two complexes different. In HCC patients, activation of the mTOR pathway was observed in 40-50% HCC patients and was closely associated with HCC poor prognosis and early recurrence [17, 20], which facilitated the development of mTOR inhibitors for clinical applications.

In this study, our analyses from TCGA dataset revealed that the majority of FOX proteins (27/40) was overexpressed in HCC patients and their expression levels were tightly correlated with tumor stage, tumor grade and overall survival of HCC patients. We also verified that FOXH1 was indeed highly expressed in our collected HCC samples compared to the corresponding adjacent tissues. And experimental results demonstrated that FOXH1 played an oncogenic role in HCC development, which was partially due to the activation of mTOR signaling. Indeed, inhibition of mTOR signaling with rapamycin could reverse FOXH1-induced HCC cell growth and cell invasion/migration. Furthermore, our *in vivo* animal data also confirmed that FOXH1 promoted HCC tumor growth. Overall, our study comprehensively analyzes the clinical values of FOX proteins in HCC and provides compelling rationale to develop FOX proteins either as diagnostic/prognostic or therapeutic biomarkers for better treatment of HCC patients.

## RESULTS

### Expression levels of FOX family members were increased in HCC patients

**Figure 1:**
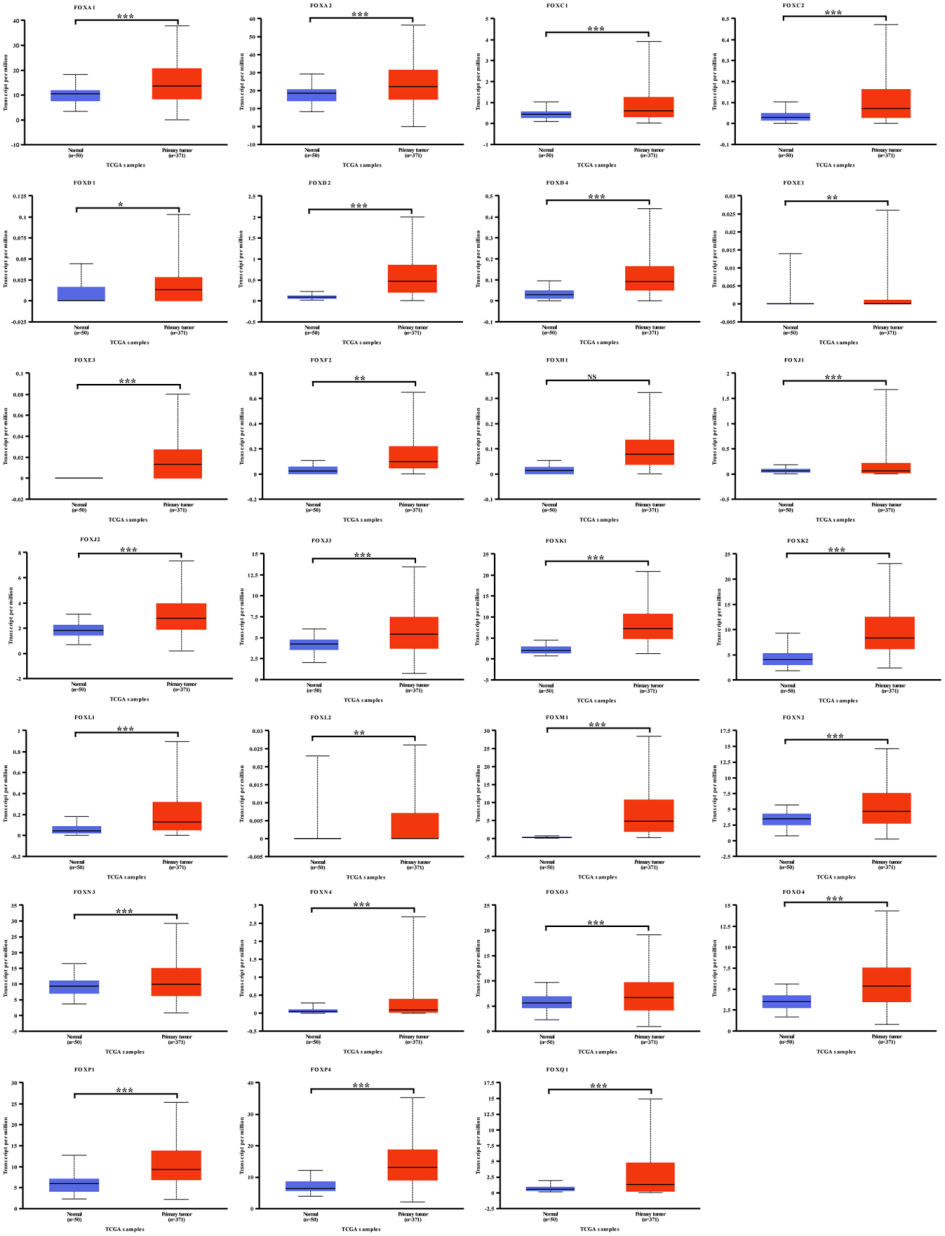
**Expression levels of FOX family members were upregulated in HCC patients.** 27 FOX proteins were upregulated in HCC patients compared to the normal liver tissues. *P<0.05, **P<0.01, ***P<0.001.

**Figure 2:**
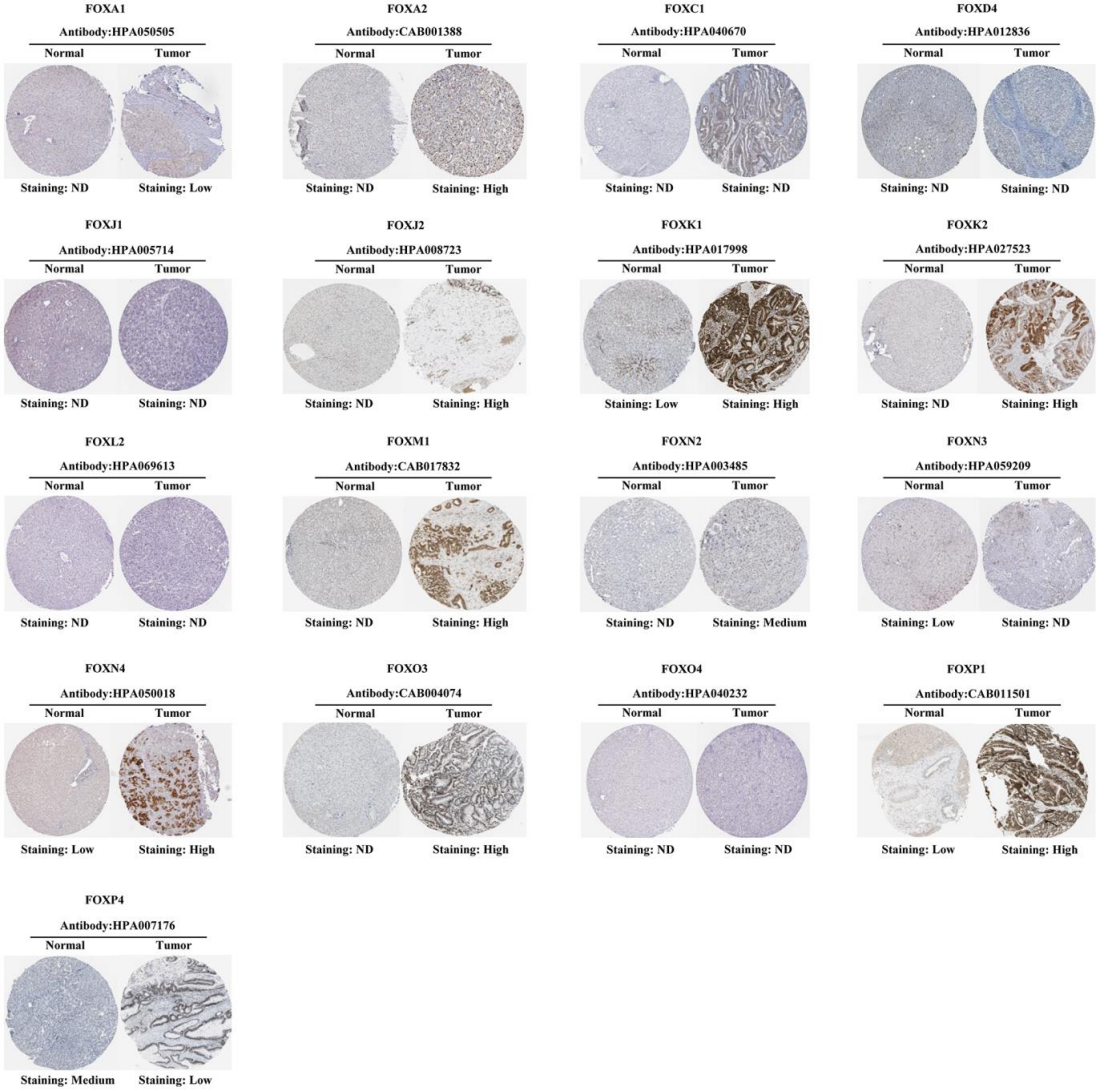
**Representative immunohistochemical images of FOX proteins in HCC samples and the normal liver tissues.** ND: Not detectable.

A lack of comprehensive analysis of FOX family members in HCC patients led us to examine their expression levels by using UALCAN (http://ualcan.path.uab.edu) online tool. As data presented in Figure 1, 27 FOX family members were highly expressed in HCC patients compared to normal liver tissues. Of note, other 13 FOX members failed to show this trend (Supplementary Figure 1): 7 FOX proteins (FOXB1, FOXB2, FOXG1, FOXI1, FOXN1, FOXR1 and FOXR2) were undetectable in both HCC and liver tissues; 4 FOX members (FOXD3, FOXF1, FOXO1 and FOXP2) were under expressed in HCC patients; FOXA3 and FOXP3 were indistinguishably expressed between HCC and normal liver tissues. Moreover, protein examination by Human Protein Atlas (https://www.proteinatlas.org) also confirmed that the majority of FOX proteins was overexpressed in HCC patients (Figure 2). Together, these analyses suggest that most FOX family proteins are highly expressed in HCC samples and they may serve as oncogenic factors to promote HCC progression.

### The expression levels of FOX proteins were independent predictors for overall survival of HCC patients

**Figure 3:**
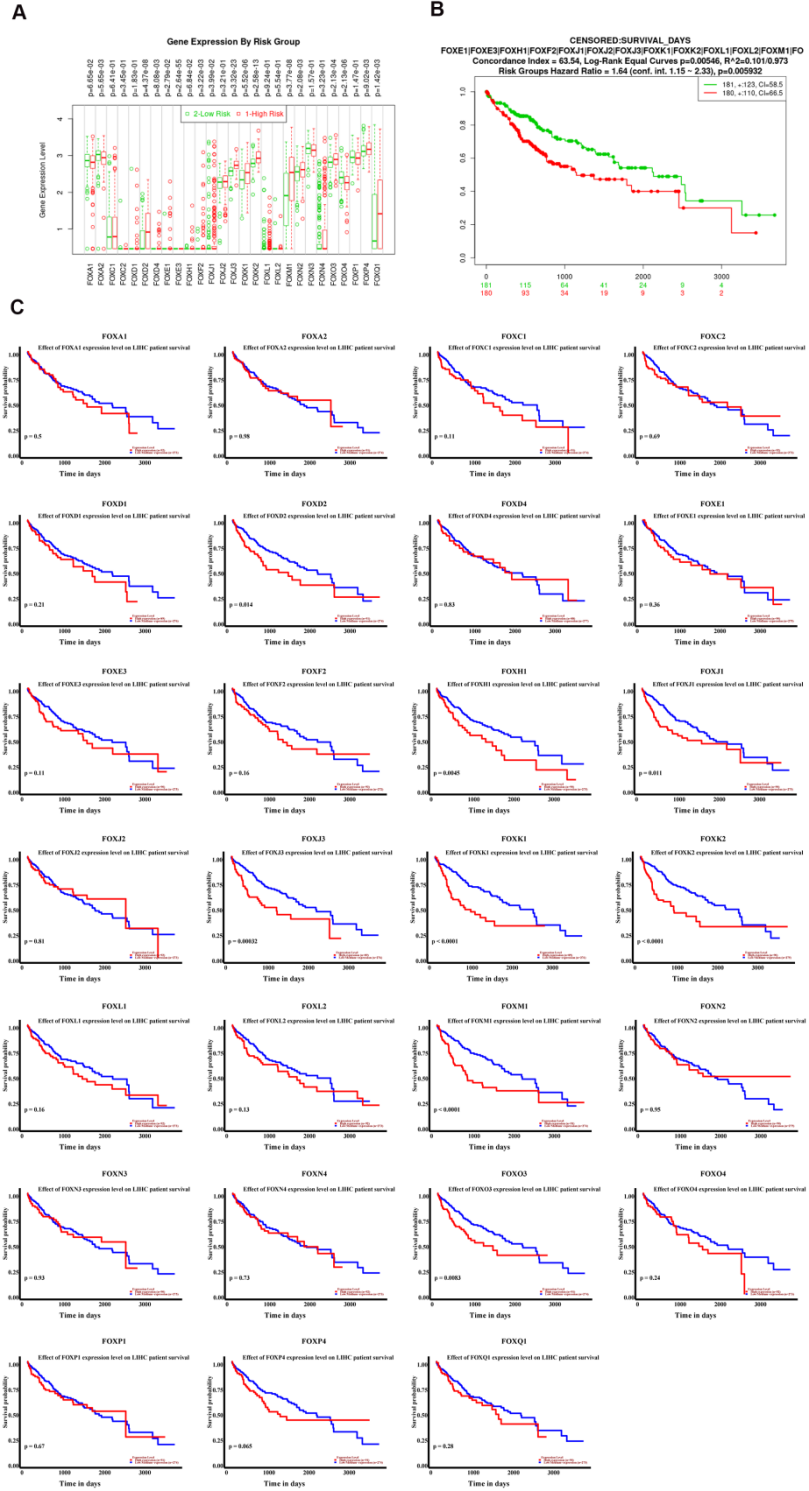
**The expression levels of FOX proteins were independent predictors for overall survival of HCC patients.** (**A**) The Box plots of individual FOX protein in low (green) and high (red) risk groups of TCGA-LIHC patients. (**B**) Cox overall survival analysis in SurvExpress showed that HCC patients with high expression levels of FOX proteins had shorter overall survival. (**C**) The association between Individual FOX protein expression level with overall survival of HCC. ^*^P<0.05, ^**^P<0.01, ^***^P<0.001.

To explore whether the expression levels of FOX proteins were associated with the prognosis of HCC, we first divided HCC patients into low-risk (n=181) and high-risk (n=180) group using the median expression level of individual FOX as a cutoff (Figure 3A). Cox overall survival analysis in SurvExpress (http://bioinformatica.mty.itesm.mx:8080/Biomatec/SurvivaX.jsp) demonstrated that high-risk group had poor overall survival compared to low risk group (Figure 3B, HR=2.16, P<0.001), indicating that the expression levels of these FOX proteins were highly associated with the overall survival of HCC. Also, the association of individual FOX protein with the overall survival of HCC was analyzed by UALCAN, which revealed that the majority of FOX proteins was highly expressed in HCC patients who had shorter overall survival time (Figure 3C). Furthermore, we also found that the expression levels of most FOX proteins were also tightly associated with tumor stage, tumor grade and metastatic status of HCC. Data showed that high grade, high stage or metastatic HCC patients tended to express high levels of FOX proteins (Supplementary Figures 2–4). Of note, although metastatic HCC expressed higher levels of FOX proteins, there was no statistically significant difference, largely due to the small sample size (n=4). Collectively, all these findings indicate that the expression levels of most FOX family members can serve as independent predictors for the prognosis of HCC patients.

### Experimental evidence suggested that FOXH1 promoted HCC development

**Figure 4:**
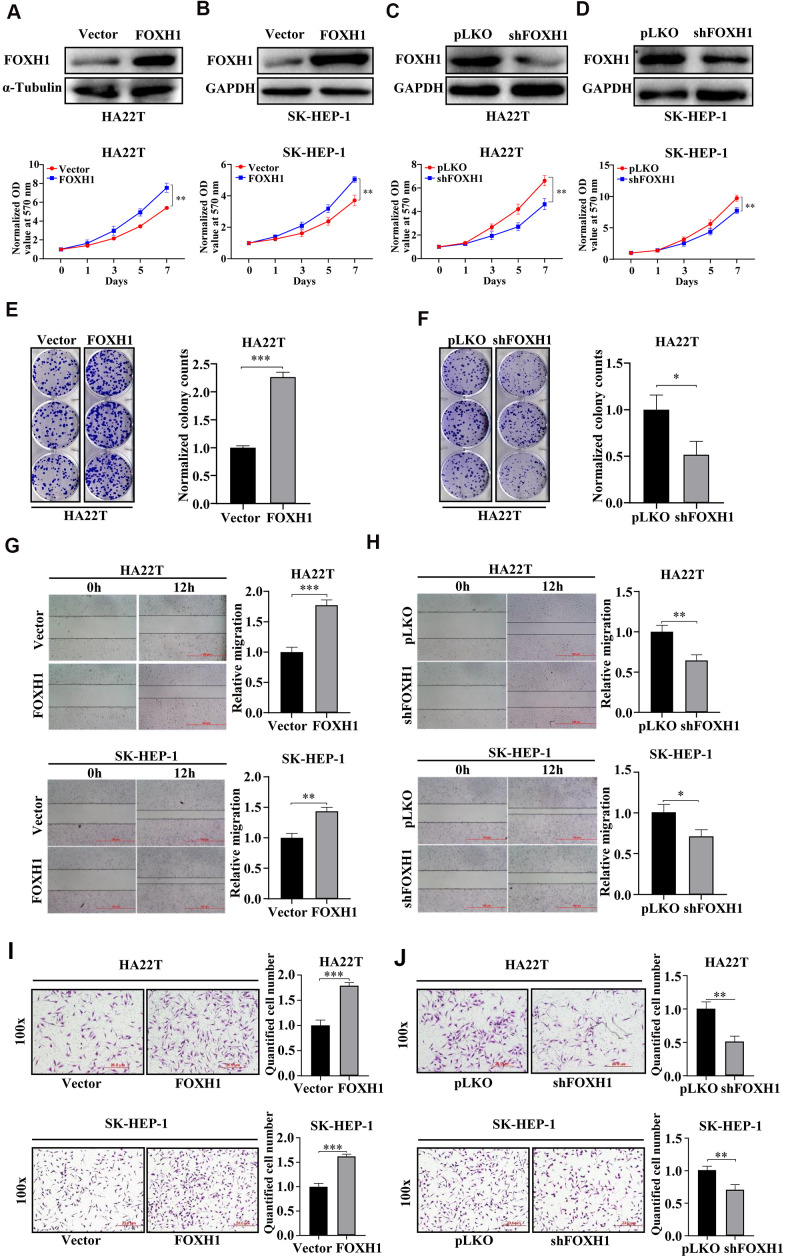
**Experimental evidence suggested that FOXH1 promoted HCC development.** (**A**, **B**) Top, the efficiency of FOXH1 overexpression. GAPDH was internal control. Bottom, MTT assay demonstrated that overexpression of FOXH1 promoted cell growth of HA22T (**A**) and SK-HEP-1 cells (**B**). (**C**, **D**) Top, efficiency of FOXH1 knockdown. GAPDH was loading control. Bottom, MTT assay revealed that knockdown of FOXH1 reduced cell growth of HA22T (**C**) and SK-HEP-1 cells (**D**). (**E**) FOXH1 HA22T cells had better colony forming ability than Vector HA22T cells. Left, representative images of colonies. Right, statistical analysis. (**F**) FOXH1 depleted HA22T cells formed less colonies than the control cells. Left, representative images of colonies. Right, statistical analysis. (**G**) Overexpression of FOXH1 increased cell migrating ability of HA22T (top) and SK-HEP-1 cells (bottom) cells. Left, representative images of wounding healing assay. Right, statistical analysis. (**H**) FOXH1 knockdown suppressed the migrating ability of HA22T (top) and SK-HEP-1 cells (bottom) cells. Left, representative images of wounding healing assay. Right, statistical analysis. (**I**) Overexpression of FOXH1 promoted cell invasion of HA22T (top) and SK-HEP-1 (bottom) cells. Left, representative images of wounding healing assay. Right, statistical analysis. (**J**) Knockdown of FOXH1 decreased cell invasion of HA22T (top) and SK-HEP-1 (bottom) cells. Left, representative images of wounding healing assay. Right, statistical analysis. ^*^P<0.05, ^**^P<0.01, ^***^P<0.001.

Next, we turned our focus on FOXH1 because its role in HCC progression has not yet been determined. The expression level of FOXH1 was tightly associated with tumor stage, tumor grade and overall survival of HCC (Figure 3C and Supplementary Figures 2–4). Indeed, overexpression of FOXH1 in HA22T promoted their growth, monitored by MTT assay (Figure 4A). Similar result was gained in SK-HEP-1 cells (Figure 4B). On the contrary, FOXH1 depletion by shRNAs suppressed cell growth of HA22T and SK-HEP-1 cells (Figure 4C, 4D). Also, our data revealed that overexpression of FOXH1 increased the colony forming ability of HA22T cells (Figure 4E) while knockdown of FOXH1 inhibited the colony forming ability of HA22T cells (Figure 4F).

Next, we sought to investigate whether FOXH1 could regulate cell migration and cell invasion of HCC cells. Results from wound healing assay indicated that FOXH1 overexpression could increase cell migrating ability of both HA22T and SK-HEP-1 cells (Figure 4G) while FOXH1 depletion led to suppressed cell migration of HA22T and SK-HEP-1 cells (Figure 4H). Consistently, data from transwell invasion assay suggested that HA22T and SK-HEP-1 cells with FOXH1 overexpression had stronger invasive abilities compared to the corresponding control cells (Figure 4I). In contrast, knockdown of FOXH1 evidently suppressed cell invasion of HA22T and SK-HEP-1 cells (Figure 4J). Taken together, these results illustrate that FOXH1 plays an oncogenic role in HCC development by promoting cell growth and cell migration/invasion.

### FOXH1 mediated cell growth and cell migration/cell invasion of HCC cells were dependent of mTOR signaling

**Figure 5:**
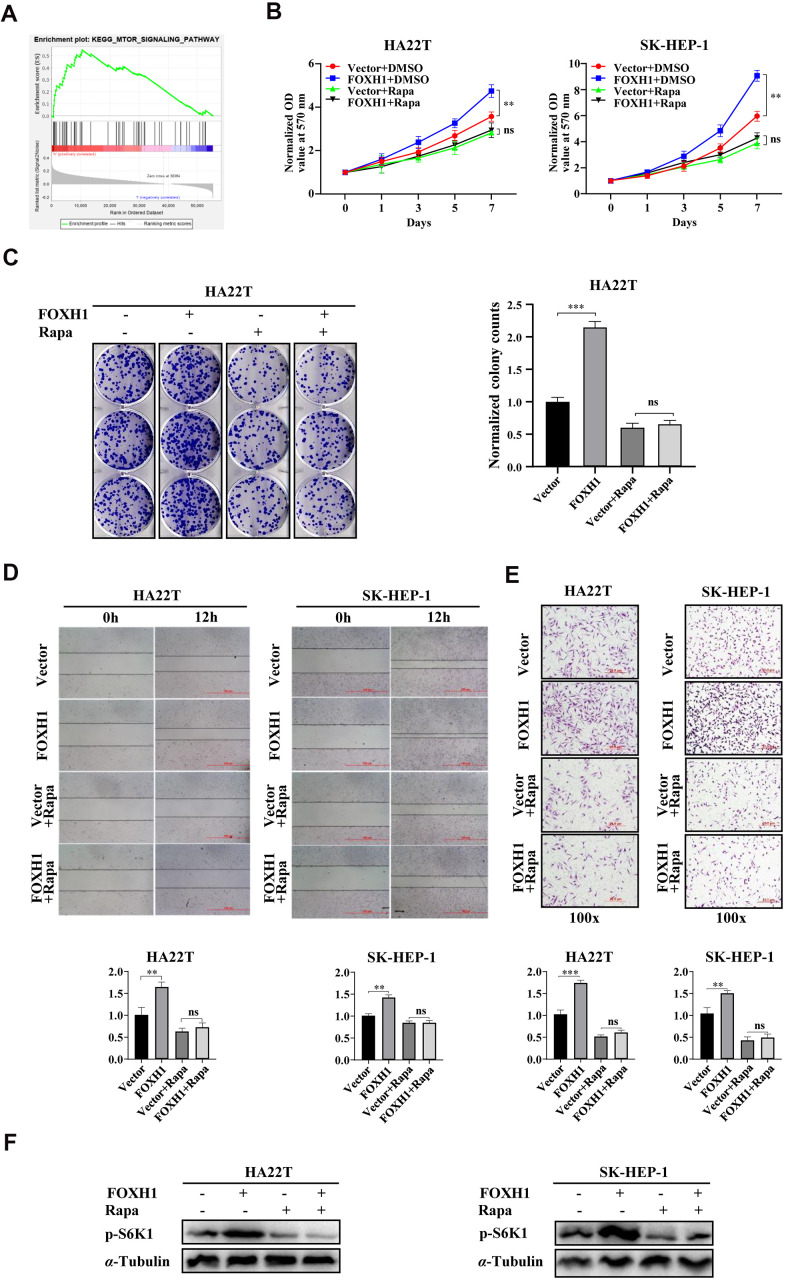
**FOXH1 mediated cell growth and cell migration/cell invasion of HCC cells were dependent of mTOR signaling.** (**A**) GSEA KEGG analysis showed that mTOR signaling was enriched in high FOXH1 HCC patients. (**B**) FOXH1 mediated cell growth of HA22T and SK-HEP-1 cells was blocked by mTOR inhibitor rapamycin (Rapa). (**C**) Colony formation assay showed that FOXH1 induced cell growth of HA22T cells was attenuated by rapamycin. Left, representative images of colonies. Right, statistical analysis. (**D**) FOXH1 lost the ability to increase cell migration of HA22T and SK-HEP-1 cells in the presence of rapamycin. Top, representative images of wounding healing assay. Bottom, statistical analysis. (**E**) FOXH1 failed to increase cell invasion of HA22T and SK-HEP-1 cells in the presence of rapamycin. Top, representative images of invading cells. Bottom, statistical analysis. (**F**) FOXH1 induced phosphorylation levels of S6K1 were blocked by rapamycin. GAPDH served as loading control. ^*^P<0.05, ^**^P<0.01, ^***^P<0.001.

To dissect the underlying mechanisms responsible for FOXH1 mediated cell growth and cell migration/invasion, we first performed GSEA KEGG pathway analysis to examine which signaling pathways were tightly associated with FOXH1 level. By dividing HCC patients into two groups (n=187 for each group) using the median expression level of FOXH1 as cutoff, we found that mTOR signaling was highly enriched in HCC patients with high level of FOXH1 (Figure 5A and Supplementary Figure 5, p=0.003). To end this, we performed the rescue assay by using mTOR specific inhibitor, rapamycin, to test whether FOXH1 mediated phenotypes of HCC cells were caused by mTOR activation. Expectedly, data revealed that FOXH1 lost its ability to increase cell growth of HA22T and SK-HEP-1 cells in the presence of 10 nM rapamycin (Figure 5B), monitored by MTT assay. Colony formation assay also showed that FOXH1 failed to increase the colony number of HA22T cells when these cells were treated with 10 nM rapamycin (Figure 5C). Moreover, inhibition of mTOR signaling with rapamycin could also reverse FOXH1 induced HCC cell migration and cell invasion of HA22T and SK-HEP-1 cells (Figure 5D, 5E). As the downstream indicator of mTOR signaling, the phosphorylation level of S6K1 was also increased by FOXH1 induction, which was attenuated by rapamycin treatment (Figure 5F). Together, all these data support the notion that FOXH1 mediated cell growth and cell migration/invasion are at least partially caused by the activation of mTOR signaling.

### *In vivo* and clinical evidence confirmed the oncogenic role of FOXH1 in HCC development

**Figure 6:**
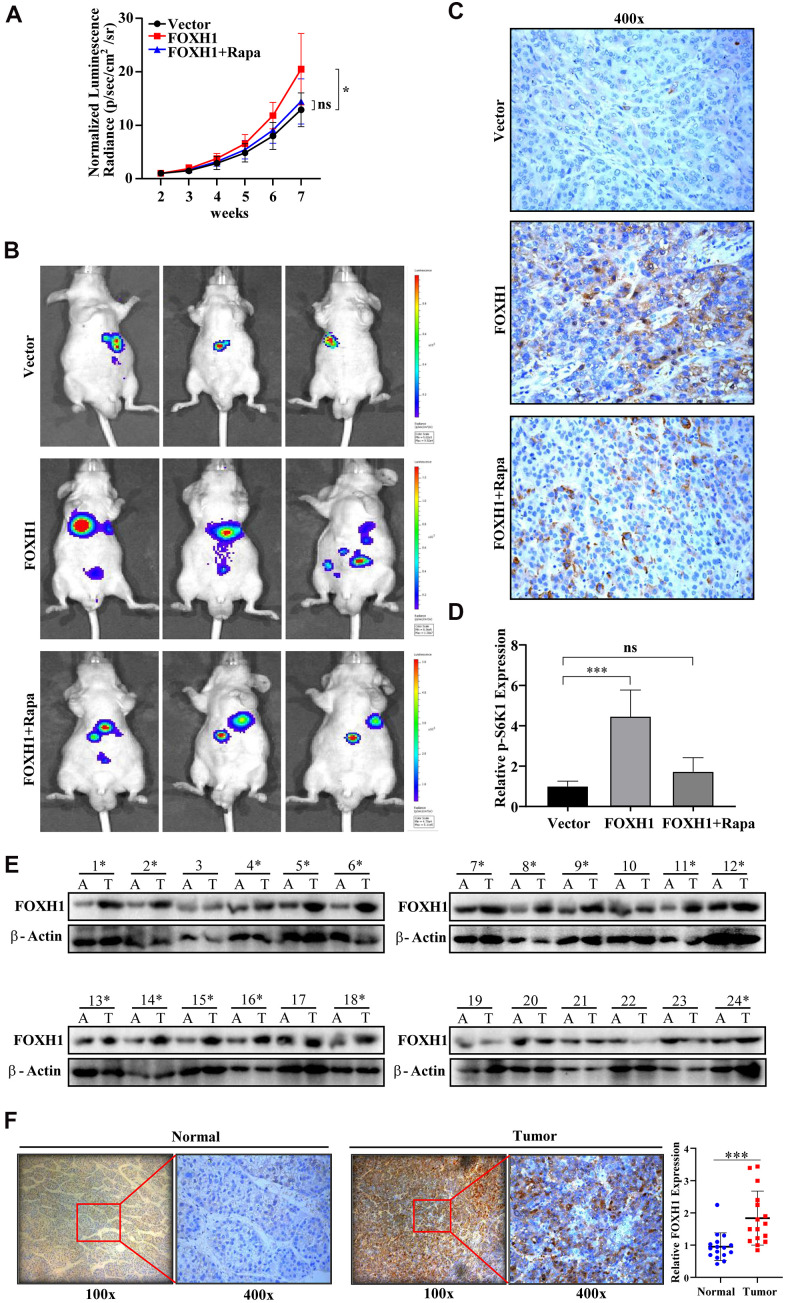
***In vivo* and clinical evidence confirmed the oncogenic role of FOXH1 in HCC development.** (**A**) The HA22T tumor growth curve. (**B**) Representative images of HA22T tumors. (**C**) p-S6K1 staining showed that FOXH1 promoted HA22T tumor growth was dependent of mTOR activation. (**D**) A statistical analysis of p-S6K1 level. (**E**) Western blotting analysis of FOXH1 in 24 HCC samples with their paired adjacent liver tissues. (**F**) IHC staining of FOXH1 in HCC samples using their corresponding adjacent liver tissues as controls. Left, representative image of FOXH1 staining in slides made from patient 1#. Right, statistical analysis of FOXH1 staining. Scale bar: 25 μm. *P<0.05, **P<0.01, ***P<0.001.

To test whether FOXH1 promoted HCC progression *in vivo*, we first orthotopically implanted luciferase-based Vector or FOXH1 HA22T (1X106) into the livers of nude mice and examined tumor growth by using IVIS system. Data showed that FOXH1 indeed could significantly promote HCC tumor growth (Figure 6A, 6B). Most importantly, FOXH1 HA22T tumors were considerably suppressed when we administrated them with 2 mg/kg rapamycin (Figure 6A, 6B), strengthening the notion that the contribution of FOXH1 to HCC progression was at least partially dependent of mTOR signaling. Immunohistochemical staining of mTOR signaling marker, p-S6K1, also confirmed that the tumor promoting role of FOXH1 in HA22T xenografted mouse model was dependent of mTOR activation (Figure 6C, 6D). Moreover, we collected HCC samples (clinical information was listed in Table 1) and the paired adjacent liver tissues to examine FOXH1 level. Consistent to our above results, high expression level of FOXH1 was observed in 16 HCC patients compared to the paired adjacent liver tissues (Figure 6E), which was also validated with immunohistochemical staining of FOXH1 in these 16 paired tissues (Figure 6F). Collectively, all these *in vivo* and clinical results demonstrate that FOXH1 serves as a tumor promoting factor controlling HCC tumor development.

**Table 1:** Clinicopathological characteristics of HCC patients (N=24).

**Patient code**	**Patient ID**	**Gender**	**Age, year**	**Tumor number**	**HBsAg**	**AFP, ng/mL**	**Liver cirrhosis**	**BCLC stage**	**TNM stage**	**Child-pugh**
1	0008155592	Male	53	Multiple	Positive	>1210	Yes	B	III	A
2	0008146382	Male	54	Single	Positive	87.05	Yes	A	I	A
3	0000841294	Male	66	Single	Positive	>1210	Yes	0	I	A
4	0003476206	Male	56	Multiple	Positive	2.37	Yes	C	III	A
5	0008271060	Male	42	Single	Positive	4.39	Yes	A	I	A
6	0004865466	Male	20	Single	Positive	111.58	Yes	0	I	A
7	0008191254	Male	72	Single	Positive	850	Yes	A	II	A
8	0008292466	Male	64	Single	Positive	1.04	Yes	A	I	A
9	0008330242	Male	21	Multiple	Positive	>800	No	B	III	A
10	0000060449	Male	70	Single	Positive	4.99	Yes	0	I	A
11	0002543983	Male	59	Multiple	Positive	243.94	No	B	II	A
12	0004975402	Male	61	Single	Positive	400.6	Yes	A	I	A
13	0008366184	Male	54	Single	Positive	1.4	Yes	A	I	A
14	0002933746	Male	66	Single	Positive	1.18	No	A	I	A
15	0008075110	Male	41	Single	Positive	4.47	No	A	I	A
16	0001982601	Male	55	Single	Positive	2	Yes	A	I	A
17	0008435226	Female	65	Single	Positive	6.36	No	A	I	A
18	0008418675	Male	64	Single	Positive	14.15	Yes	A	I	A
19	0008464942	Male	48	Single	Positive	602.95	Yes	A	I	A
20	0008440382	Male	57	Single	Positive	>1210	Yes	A	I	A
21	0008465012	Male	50	Single	Positive	56.71	No	A	I	A
22	0003711350	Male	65	Single	Positive	>1210	Yes	A	I	A
23	0008509448	Male	56	Single	Positive	230.41	Yes	0	I	A
24	0008560310	Male	49	Multiple	Positive	9.92	No	B	III	A

## DISCUSSION

Identification of novel diagnostic/prognostic or therapeutic biomarkers of HCC is still one of scientific focuses. In this study, we comprehensively analyzed the potential clinical value of individual FOX protein in HCC by using TCGA-LIHC dataset. Our data revealed that the majority of FOX proteins was highly expressed in HCC patients using the normal liver tissues as controls. Also, higher expression levels of FOX proteins were associated with higher stage, higher metastasis and shorter survival time of HCC. Importantly, our experimental confirmation using FOXH1 as example suggested that FOXH1 indeed promoted HCC progression by triggering mTOR activation. Our study not only pinpoints the clinical value of each FOX protein in HCC but also strengthens the tumor promoting role of FOXH1 with both experimental and clinical evidence. Therefore, we believe development of FOX targeted therapeutic approaches may improve current treatments of HCC.

Recently, accumulating evidence suggested that FOX proteins were involved in cancer carcinogenesis. Immunohistochemical staining of FOXO3 demonstrated that FOXO3 was overexpressed in HCC samples, suggesting it may occupy a position in HCC development. Indeed, hepatic FOXO3a transgenic mice were much easier to develop tumorigenesis upon hepatotoxicin treatment compared to the control cohorts [21]. Study also documented that FOXG1 was highly expressed in HCC patients and its induction could promote EMT (epithelial-Mesenchymal transition) by activating beta-catenin signaling [11]. Moreover, the roles of FOXK1 and FOXQ1 in HCC in progression have also been well documented. An increased expression level of FOXK1 caused by DNA hypomethylation was observed in HCC patients [15]. FOXK1 upregulation in HCC cells led to expand the population of cancer stem cells by increasing EpCAM and ALDH1 expression levels [15]. FOXQ1 upregulation could initiate HCC by enhancing the communication between cancer association fibroblasts (CAF) and tumor cells [22]. Our analyses of FOXK1, FOXQ1, FOXG1 and FOXO3a in TCGA LIHC dataset were consistent with previous findings. To summarize, the roles of FOX members in HCC initiation and progression are increasingly discovered. In this study, a comprehensive analysis of FOX members in HCC demonstrates that some FOX family members could serve as prognostic factors determining HCC progression.

Indeed, experimental validation demonstrated that FOXH1 promoted cell growth and cell invasion/migration of HCC cells. The *in vivo* animal data also confirmed the oncogenic role of FOXH1 in HCC development. Mechanistically, FOXH1 mediated HCC cell growth and cell invasion was partially caused by mTOR activation. As fact, the role of mTOR signaling in HCC progression has been well documented. Clinical data showed that mTOR signaling was activated in an approximate 40% HCC patients [16, 17, 20], facilitating the clinical application of mTOR inhibitors into HCC treatment. Although mTOR inhibitors such as everolimus, temsirolimus and deforolimus have been approved for the treatment of RCC (renal cell carcinoma), HER2-negative breast cancer and pancreatic neuroendocrine tumor [23–25], they haven’t obtained approval for treatment in HCC patients. Nevertheless, mTOR inhibitors have been showed to suppress HCC cell growth *in vitro* and *in vivo*. Most importantly, they are being clinically tested for the treatment of advanced HCC. Our results revealed that mTOR inhibitor rapamycin could block FOXH1 induced HCC cell growth *in vitro* and *in vivo*. All these facts build rationale to apply mTOR inhibitor for the treatment of HCC patients with high FOXH1 expression level.

In addition, we found that metastatic HCC patients preferred to highly express FOX proteins even though there was no statistically significant difference due to the small size of metastatic HCC patients (n=4), indicating FOX proteins may play critical roles in the development of HCC metastasis. Metastatic HCC is more lethal compared to localized HCC so that currently therapeutic options are less effective [26]. Our data showed that FOXH1 could enhance HCC cell migration and cell invasion, two essential steps during tumor metastasis, indicating targeting FOXH1 may overcome HCC metastasis. However, how to specifically target FOXH1 is still a scientific question. Fortunately, specific inhibitors towards FOX proteins were recently being developed. JBIR-141 and JBIR-142 have been identified as potent inhibitors specific for FOXO3a [27]. Given that FOX proteins are increased in metastatic HCC, it is worthy to develop their specific inhibitors for better treatment of this type of HCC.

Overall, our study comprehensively analyzed the correlations between the expression levels of FOX proteins and the clinical characteristics of HCC, pinpointing the prognostic values of FOX proteins in HCC progression. The experimental evidence also suggested that FOXH1 was truly a tumor promoting factor in HCC progression, strengthening the accuracy of our online analyses.

## MATERIALS AND METHODS

### Patient samples

HCC samples (24) and the paired adjacent liver tissues (24) were obtained from Department of Liver Surgery, Xiangya Hospital, Central South University. Resected tissues were stored in liquid nitrogen for further use. Informed consent was obtained from patients and study was approved by the Institutional Review board of Central South University.

### Cell culture

HA22T, SK-HEP1 and 293T were purchased from Cell Bank in Chinese Academy of Sciences (Shanghai, China). 10% FBS DMEM (100 units/mL penicillin, 100 μg/mL streptomycin) was used to culture cells in humidified 5% CO_2_ environment at 37° C.

### Lentivirus generation

PLKO with shRNAs against FOXH1 or PWPI with FOXH1 cDNA was co-transfected with psPAX2 and pMD2.G into 293T cells using the standard calcium phosphate transfection method. 48 hours post-transfection, virus supernatant was collected and infected HCC cells. 8 μg/ml polybrene was used to enhance the efficiency of virus infection.

### Real time qPCR

Total RNAs were isolated using Trizol reagent following the standard RNA extraction protocol. Reverse transcription was performed using two μg of total RNAs. Real time qRT-PCR was performed using a Bio-Rad CFX96 system with SYBR green to determine the mRNA expression levels of interested genes. GAPDH mRNA level was served as internal control.

### Western blotting

Lysates from HA22T, SK-HEP1 cells were loaded into 10%-12% SDS-PAGE gel for electrophoresis before proteins were transferred to PVDF membrane. After being blocked with 10% milk in TBST, membrane was probed with specific primary antibody with approximate dilution at 4° C for at least 16 hours, followed by incubation with 1:5000 HRP conjugated secondary antibody for 1 hour at room temperature. After being washed with TBST, blots were analyzed using enhanced chemiluminescence. Anti-FOXH1 (ab189960, Abcam) and anti-GAPDH (sc-47724, Santa Cruz) were used in this study.

### MTT cell growth assay

HA22T, SK-HEP1 cells were seeded into 24-well plates at a density of 5000 cells/well. Cells at different time points were incubated with 5 μg/ml 3-(4,5-Dimethylthiazolyl)-2,5-diphenyltetrazolium bromide (MTT, Abcam) for 3 hours, followed by 10 mins DMSO incubation and the absorbance was analyzed at 490 nm.

### Wound healing assay

HA22T, SK-HEP1 cells with or without FOXH1 manipulation were scratched using a pipette tip. 48 hours later, images of these cells were taken by microscopy.

### Transwell assay

HA22T, SK-HEP1 cells with or without FOXH1 manipulation were loaded into the upper chamber of the inserts pre-coated with 1:8 diluted matrigel (BD Bioscience, USA) at the density of 1 × 10^5^/well. 0% FBS medium in the lower chamber served as a chemoattractant. 24 hours later, the invading cells in inserts were stained with 0.1% crystal violet and imaged with inverted microscope (Olympus, Japan). Quantification of images was done with ImageJ.

### *In vivo* xenografted mouse model

1X10^6^ HA22T-luciferase cells were orthotopically implanted into nude mice to allow tumor growth. Tumors were measured by IVIS system every week. 4 weeks post-implantation, mice were sacrificed and tumors were removed for IHC staining.

### Immunohistochemical staining (IHC)

Tissues were fixed in 10% (v/v) formaldehyde in PBS and embedded in paraffin. 5 μm sections were made and treated with boiling citrate buffer (pH 6.0) for 30 mins to complete antigen retrieval. After incubation with 3% peroxidase and 10% goat serum blocking buffer, the slides were incubated with 1:100 diluted anti-FOXH1 (ab189960, Abcam) or anti-p-S6K1 (9234, CST) primary antibody at 4° C for at least 16 hours. Then the slides were incubated with biotin-labeled secondary antibody for another 30 min, and followed by 30 min streptavidin incubation (PK-4000, Vectastain, USA.) The FOXH1 signal was determined by DAB staining.

### GSEA KEGG pathway analysis

The mRNA profile in TCGA-LIHC and the corresponding clinical information were downloaded from Xena Functional Genomics Explorer (https://xenabrowser.net/heatmap/) of the University of California Santa Cruz. KEGG pathway analysis was performed using GSEA online tool (http://www.broadinstitute.org/gsea) after dividing patients into two groups using the median expression level of FOXH1.

### Statistical analysis

All statistical analyses were performed using GraphPad Prism software. Data were presented as mean ± SE. Differences were analyzed with the Student t test, and significance was set at P <0.05. *, ** and *** indicates P < 0.05, P < 0.01 and P<0.001, respectively.

## Supplementary Material

Supplementary Figures
