# Supplementary material for: A comprehensive analysis of FOX family in HCC and experimental evidence to support the oncogenic role of FOXH1
Source: Aging (Albany NY). 2022 Mar 7;14(5):2268–86. doi: 10.18632/aging.203934 (PMC8954963; doi:10.18632/aging.203934)
Supplement: Supplementary Figures [file aging-14-203934-s001.pdf]

SUPPLEMENTARY FIGURES

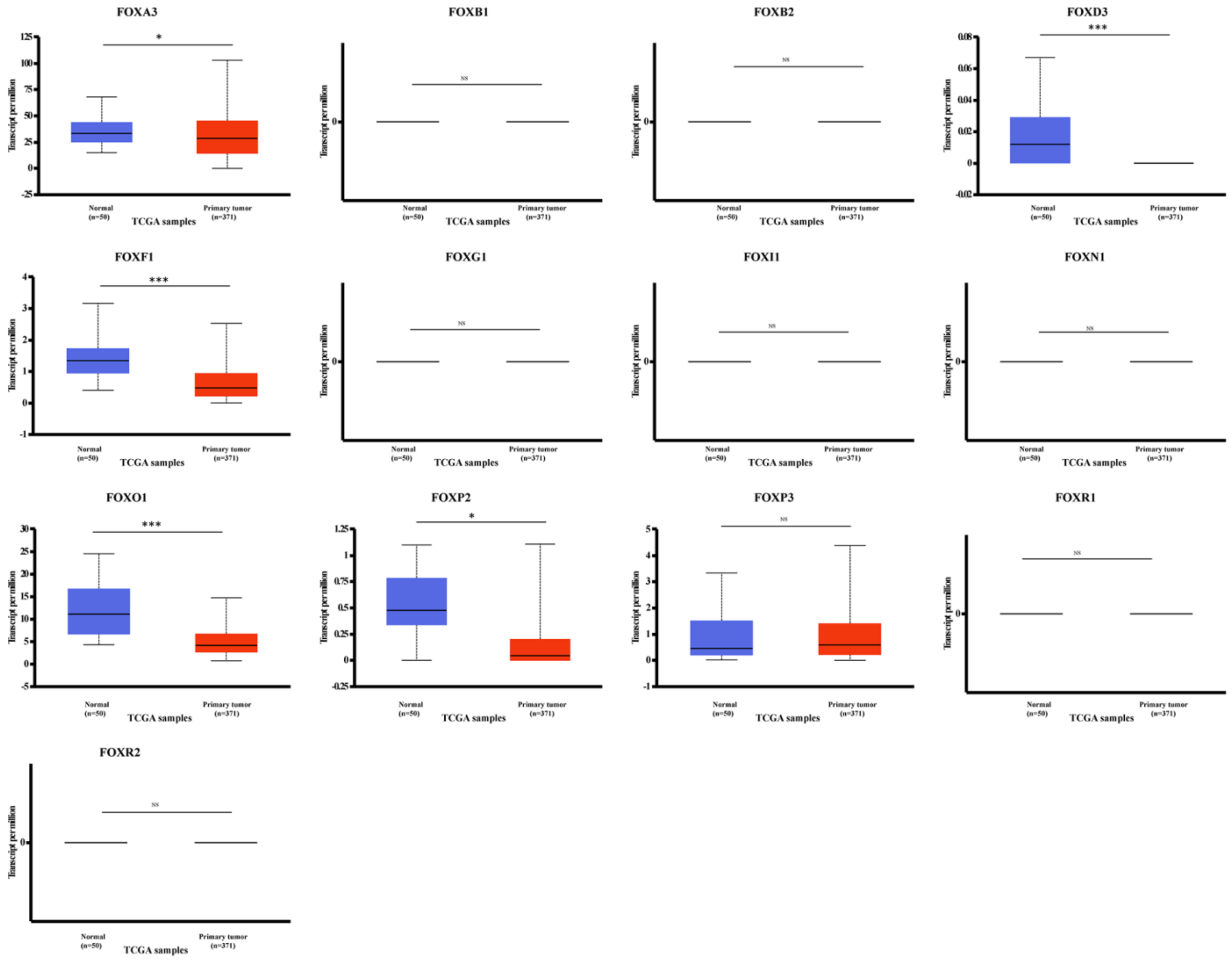

Supplementary Figure 1. 13/40 FOX proteins were not upregulated in HCC patients compared to the normal liver tissues.

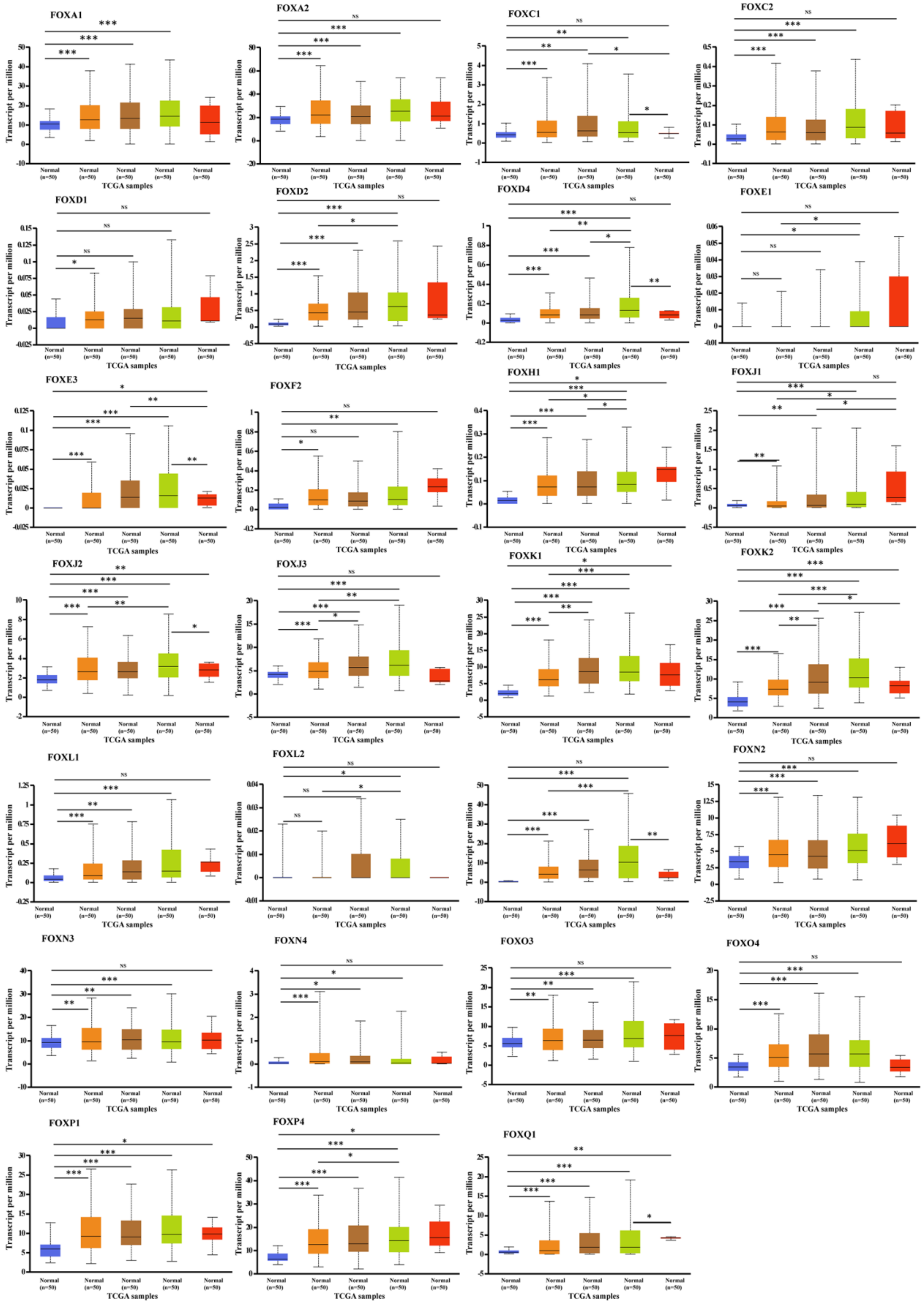

Supplementary Figure 2. The association of individual FOX mRNA level with tumor stage of HCC.

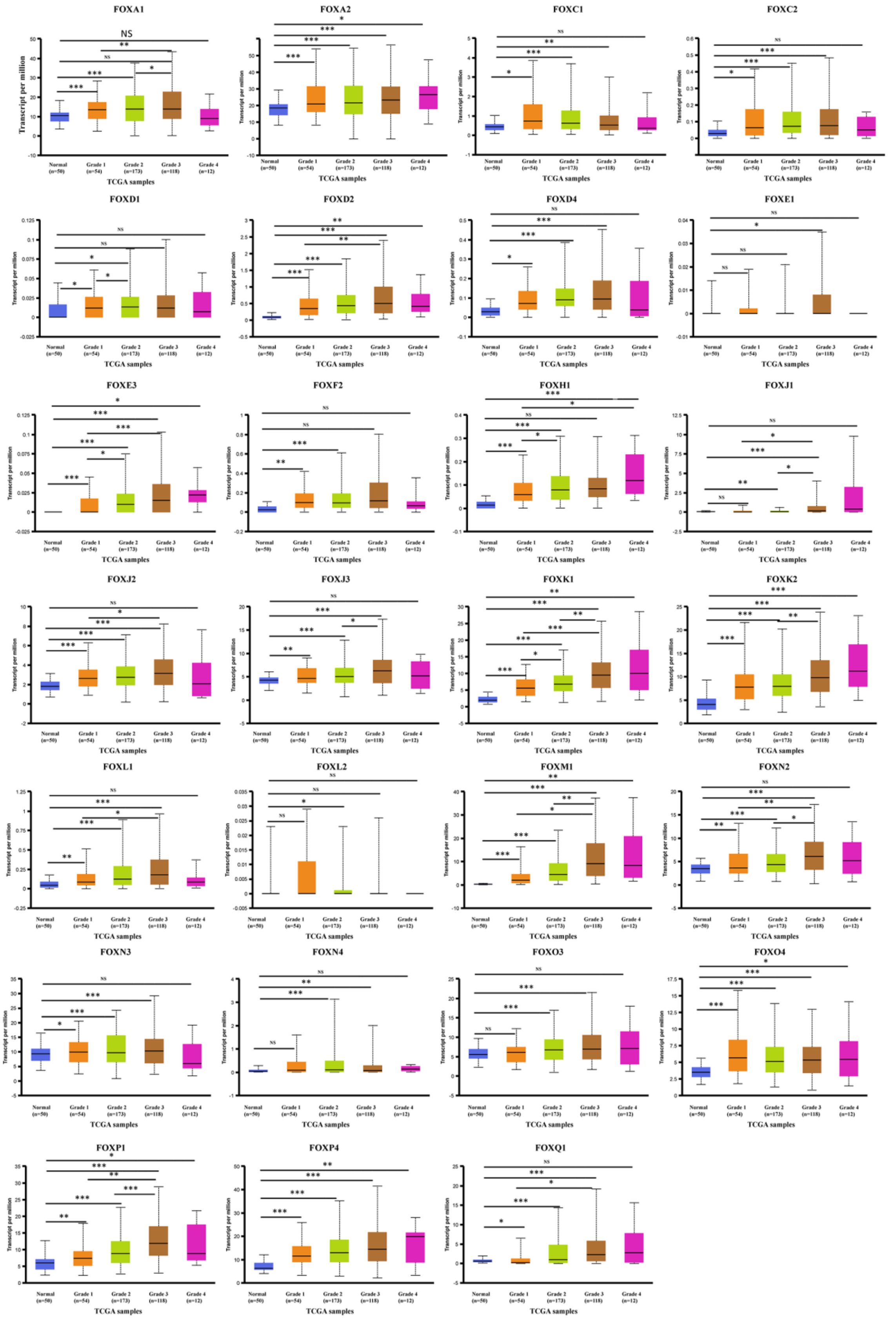

Supplementary Figure 3. The association of individual FOX mRNA level with tumor grade of HCC. *P<0.01, **P<0.01, ***P<0.001.

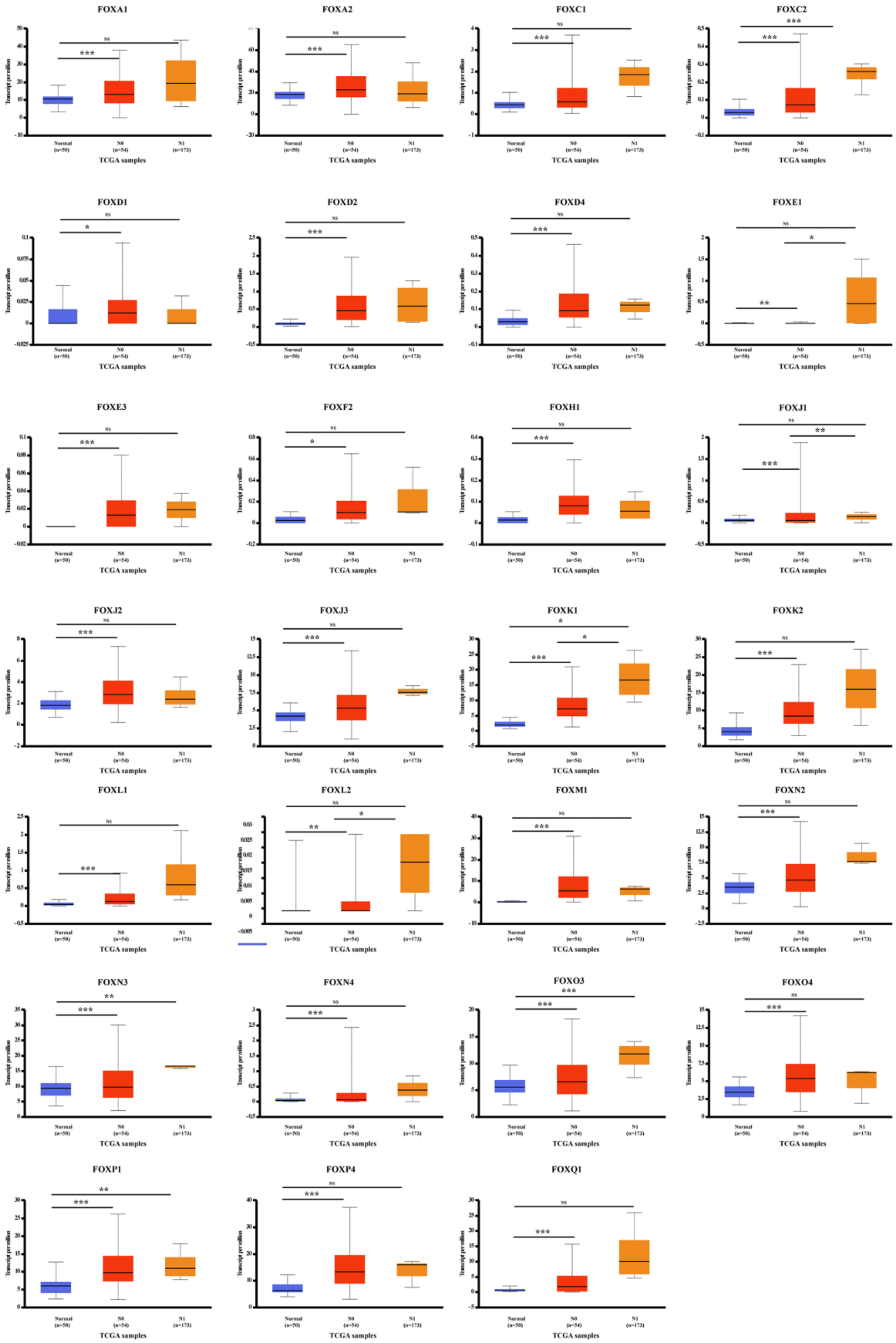

Supplementary Figure 4. The association of individual FOX mRNA level with tumor metastasis of HCC. *P<0.01, **P<0.01, ***P<0.001. NS, no significant difference.

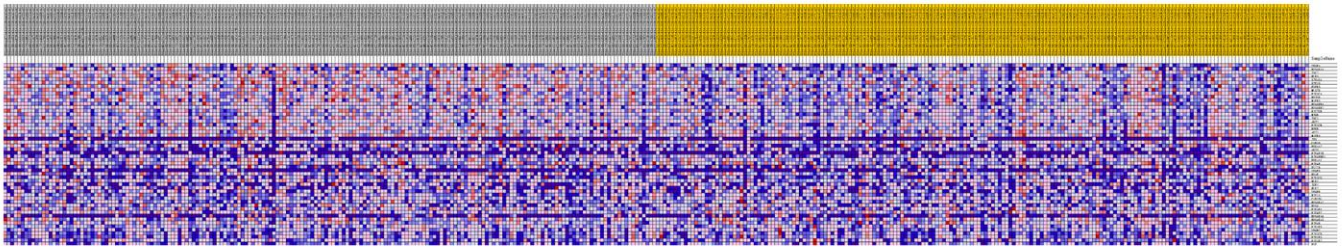

Supplementary Figure 5. Expression heatmap of mTOR signaling related molecules in high FOXH1 and low FOXH1 HCC patients.
